# The Importance of N186 in the Alpha-1-Antitrypsin Shutter Region Is Revealed by the Novel Bologna Deficiency Variant

**DOI:** 10.3390/ijms22115668

**Published:** 2021-05-26

**Authors:** Riccardo Ronzoni, Ilaria Ferrarotti, Emanuela D’Acunto, Alice M. Balderacchi, Stefania Ottaviani, David A. Lomas, James A. Irving, Elena Miranda, Annamaria Fra

**Affiliations:** 1 UCL Respiratory and the Institute of Structural and Molecular Biology, University College London, London WC1E 6JF, UK; d.lomas@ucl.ac.uk (D.A.L.); j.irving@ucl.ac.uk (J.A.I.); 2 Pneumology Unit, Centre for Diagnosis of Inherited Alpha-1 Antitrypsin Deficiency, Department of Internal Medicine and Therapeutics, IRCCS San Matteo Hospital Foundation, University of Pavia, 27100 Pavia, Italy; i.ferrarotti@smatteo.pv.it (I.F.); a.balderacchi@smatteo.pv.it (A.M.B.); s.ottaviani@smatteo.pv.it (S.O.); 3 Department of Biology and Biotechnologies ‘Charles Darwin’, Sapienza University of Rome, 00185 Rome, Italy; emanuela.dacunto@uniroma1.it (E.D.); mariaelena.mirandabanos@uniroma1.it (E.M.); 4 Italian Pasteur Institute—Cenci Bolognetti Foundation, Sapienza University of Rome, 00185 Rome, Italy; 5 Department of Molecular and Translational Medicine, University of Brescia, viale Europa 11, 25123 Brescia, Italy

**Keywords:** liver storage disease, alpha-1-antitrypsin deficiency, endoplasmic reticulum, protein aggregation, *SERPINA1* alleles, serpinopathies

## Abstract

Alpha-1-antitrypsin (AAT) deficiency causes pulmonary disease due to decreased levels of circulating AAT and consequently unbalanced protease activity in the lungs. Deposition of specific AAT variants, such as the common Z AAT, within hepatocytes may also result in liver disease. These deposits are comprised of ordered polymers of AAT formed by an inter-molecular domain swap. The discovery and characterization of rare variants of AAT and other serpins have historically played a crucial role in the dissection of the structural mechanisms leading to AAT polymer formation. Here, we report a severely deficient shutter region variant, Bologna AAT (N186Y), which was identified in five unrelated subjects with different geographical origins. We characterized the new variant by expression in cellular models in comparison with known polymerogenic AAT variants. Bologna AAT showed secretion deficiency and intracellular accumulation as detergent-insoluble polymers. Extracellular polymers were detected in both the culture media of cells expressing Bologna AAT and in the plasma of a patient homozygous for this variant. Structural modelling revealed that the mutation disrupts the hydrogen bonding network in the AAT shutter region. These data support a crucial coordinating role for asparagine 186 and the importance of this network in promoting formation of the native structure.

## 1. Introduction

Endoplasmic reticulum storage diseases (ERSDs) are a group of genetically based disorders in which mutant proteins tend to accumulate in the endoplasmic reticulum (ER) as cytotoxic aggregates [1]. A representative case of liver ERSD [2] is alpha-1-antitrypsin deficiency (AATD), caused by intra-hepatic polymerization of the Z (Glu342Lys) mutant of AAT [3]. Due to chronic accumulation of intracellular polymers, ZZ homozygotes show increased risk of liver disease, that may manifest as jaundice and hepatitis in infants, and more frequently in adults as fibrosis, cirrhosis, and increased risk of hepatocarcinoma [4,5,6].

AAT is the most abundant serine protease inhibitor (serpin) in the plasma and one of the most abundant proteins produced by hepatocytes. Normal plasma levels are maintained constitutively in the 1–2 g/L range, but cytokines released during acute inflammation may increase hepatocyte production of AAT by up to 3–4 fold. The primary physiological function of AAT is the inhibition of neutrophil elastase and proteinase-3 that are released by neutrophil granulocytes during inflammation. Unbalanced protease activity in AATD results in progressive damage to the lung parenchyma that manifests with a decline of respiratory capacity in adults. The protease inhibition mechanism of AAT is based on its metastable serpin structure and on a characteristic conformational change that irreversibly inactivates the target protease [7,8]. The conformational metastability required for its physiological function renders AAT particularly vulnerable to amino acid substitutions, such as that of Z AAT, that cause folding alterations in the ER and population of a polymerization-prone intermediate conformation [9,10,11]. A fraction of the misfolded protein is degraded by ER-associated pathways [12,13], but a significant proportion escapes quality control and forms linear polymers by a domain swap mechanism [3,14,15]. Deposition of these polymers gives rise to the enlarged cisternae and inclusion bodies that are detected histologically by PAS (periodic acid-Schiff)-diastase staining and have been characterized morphologically by electron microscopy [3,15]. Polymers of Z AAT have also been found in the circulation [16] and in the lung interstitium [17], where they are thought to stimulate chemotactic recruitment and degranulation of neutrophils, thus amplifying tissue damage of the lung parenchyma and the progression to emphysema [18]. Extracellular polymers have been shown to derive from active cell secretion rather than being released by cell death or formed by extracellular polymerization of secreted monomeric protein [19,20].

Compared to Z AAT, which reduces circulating AAT to around 15% in ZZ homozygotes, the S AAT variant (Glu264Val) shows a milder secretion defect and a much lower tendency to polymerize [21]. Accordingly, SS homozygotes rarely show lung or liver disease, while both manifestations are frequently observed in SZ compound heterozygotes [6,22]. This likely results from SZ heteropolymerization, as demonstrated in cellular models of disease [23] and in circulating polymers [24]. Besides the relatively common S and Z AAT mutants, more than 50 ultrarare missense variants have been found in AATD patients, generally in heterozygosity with Z or S AAT [25,26]. A subset of them have been reported to cause liver disease, but due to their rarity and the paucity of epidemiological data, their polymerogenic profile has been mainly revealed through in vitro studies or by expression in cellular models. Many of the polymerogenic mutations identified so far cluster in the core shutter region of the AAT molecule that regulates opening of β-sheet A to allow insertion of the reactive centre loop as β-strand 4 [27]. Within this group, we find the Mmalton and Mpalermo variants that carry the same single amino acid deletion (Phe51del), respectively on the M1 and M2 haplotype backgrounds. The alleles encoding these variants have been identified in several AATD cases across Southern European and North African countries [28]. Studies focused on the clinical profiles of Mmalton carriers have showed marked plasma deficiency, severe pulmonary manifestations, liver inclusion bodies and hepatic disease [29,30,31,32,33]. Accordingly, Mmalton AAT showed high polymerogenicity in vitro [32,34]. A similar molecular change causes polymer formation by Siiyama (Ser53Phe) AAT, an ultrarare variant identified in Japan [35,36,37]. Another shutter domain variant, King’s AAT (His344Asp), showed a high degree of polymer formation, intracellular retention, and higher presence of polymers in the culture media of transfected cells when compared to Z AAT [38].

We report here a novel amino acid substitution identified in the shutter region of AAT, which was found in five unrelated subjects and named Bologna. We present the clinical profiles of the subjects bearing the novel mutation and the characterization of the Bologna protein in cellular models in comparison with the polymerogenic Z, Mmalton and King’s AAT variants. Finally, we describe, with reference to the native AAT crystal structure, that the effects we see are most likely the result of disruption of an important hydrogen bond network within the AAT shutter region.

## 2. Results

### 2.1. Identification and Clinical Profile of the Bologna AAT Variant

The new AAT allele was identified for the first time in 2013, at the Reference Centre in Pavia (Italy), in a 50-year-old male (Table 1, subject 1.1), previous smoker (25 pack/years) suffering from centrilobular emphysema, bronchiectasis, and diabetes. He also had features of mild liver steatosis. His spirometry values showed obstructive defects with pre-bronchodilator FEV1 (forced expiratory volume in 1 s) of 1.70 L (47% of predicted), and FVC (forced vital capacity) of 3.47 L (77% of predicted). His DLCO (diffusion lung carbon monoxide) was reduced (45% of predicted). The AAT plasma level was 0.23 g/L, below the so-called “protective threshold” [39] and consistent with a severe deficiency. Genetic analysis of the *SERPINA1* gene encoding AAT revealed the presence of the Z mutation in heterozygous association with a novel missense mutation in exon 2 (c.628A>T) on an M1Ala background, encoding a non-synonymous asparagine to tyrosine substitution at position 186 (N186Y; p.N210Y according to HGVS nomenclature). This AAT variant was designated “Bologna”, according to the birthplace of the proband. AAT phenotyping by isoelectric focusing (IEF) was consistent with a Z phenotype, whereas the Bologna variant was not detectable and therefore we did not assign any IEF letter designation to this novel variant. Family screening revealed the transmission of the Z and Bologna alleles from proband 1.1 to his son (subject 1.2) and daughter (subject 1.3), respectively, who were both non-smokers and healthy. The AAT plasma level was 1.49 g/L in the daughter, whose C-reactive protein levels were higher than normal, indicating the presence of an inflammatory state. After diagnosis, the proband began receiving replacement therapy by weekly intravenous infusion of exogenous AAT.

The Bologna allele was later detected in homozygosity in two unrelated subjects, both born in Egypt: (i) a 44-year-old male (subject 2.1), previous smoker (30 pack/years), metalworker, suffering from centrilobular emphysema (FEV1 54%, FVC 62% and DLCO 45% of predicted values), with a plasma AAT concentration of 0.34 g/L; and (ii) a 39-year-old male (subject 3.1), current smoker (56 pack/years), bricklayer, suffering from emphysema, with a plasma AAT level of 0.28 g/L.

Finally, the Bologna allele was detected in heterozygosity with the M1 allele in two unrelated subjects (Table 1, probands 4.1 and 5.1): (i) a 74-year-old woman, non-smoker, living in Southern Italy, reporting chronic bronchitis and emphysema, with 0.95 g/L AAT in plasma; and (ii) a 58-year-old woman, born in Ukraine, non-smoker, with no lung impairment but reporting features of hepatitis, with 0.86 g/L AAT in her plasma.

### 2.2. Structural Localization of the Bologna Mutation in the AAT Shutter Region

**Figure 1:**
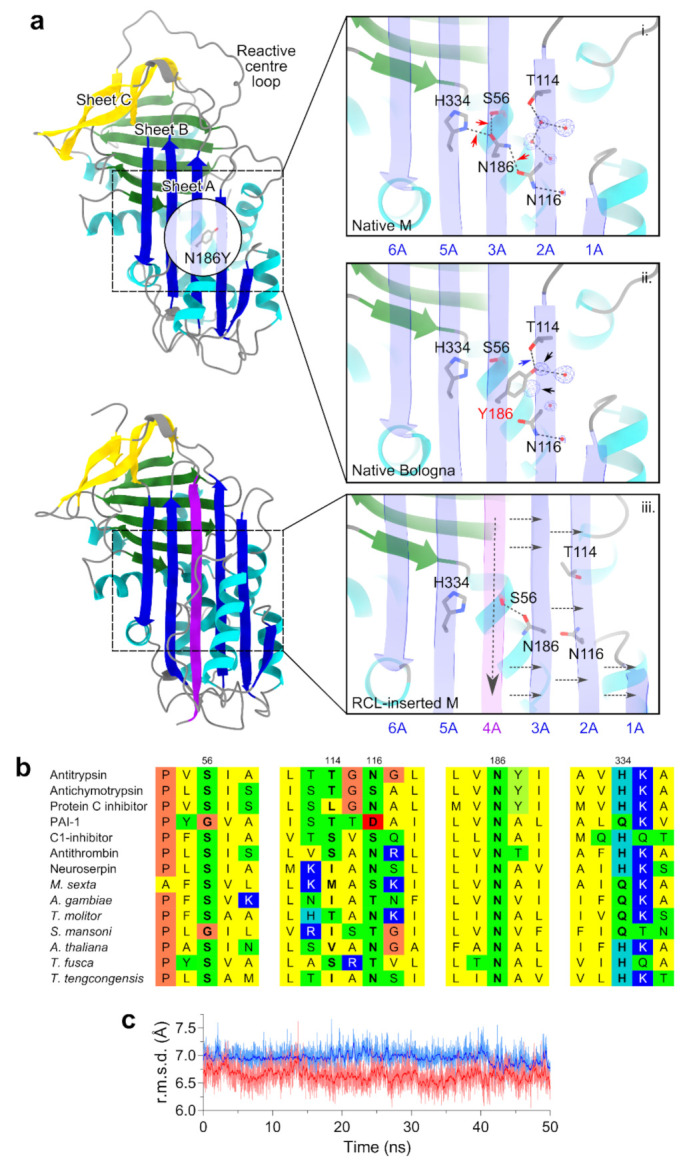
The location and predicted structural consequences of the N186Y mutation. (**a**) The introduced tyrosine at residue 186 is located in the central shutter region of β-sheet A, on strand 3, and buried into the core of the protein, as displayed in the upper left with reference to PDB structure 3NE4 [41]. This side-chain is facing away from the viewer in the orientation shown in the left panel in which obscuring elements have been made transparent in the circular ‘cut-through’. Positions of the other β-sheets and the reactive centre loop are indicated. The right panels show a close-up of the shutter region; the molecule is in an identical orientation to the representation on the left, with the helix F removed and β-sheet A made transparent for visualization purposes. In the top panel (i), the network of hydrogen bonds mediated by the side-chain of N186 are evident as dashed black lines. Water molecules appear as small red spheres; the 2Fo-Fc experimental electron density supporting their positions, calculated using the PHENIX software package [42], is shown as blue mesh and contoured at 1.0σ. Red arrows indicate the hydrogen bonds that are lost upon mutation to tyrosine. In the middle panel (ii), the further consequences of this substitution are shown: displacement of two ordered water molecules (denoted by black arrows), and formation of a novel hydrogen bond to the side-chain of T114 (blue arrow). At the lower left is the structure of cleaved AAT, 1EZX, with the reactive centre loop incorporated as an extra strand [8] (purple) and in the lower right panel (iii) the consequences of this insertion (pink) on the shutter are indicated, with dashed arrows denoting the direction of movement of structural elements. This figure was prepared using ChimeraX [43]. The β-sheet A strand designations are shown between panels (i) and (ii) and below panel (iii). (**b**) The high degree of conservation of asparagine at position 186 and of histidine or glutamine at 334 is evident from a structure-based sequence alignment of serpins from different branches of the tree of life, based on mammalian antitrypsin (PDB accession 3NE4), antichymotrypsin (1YXA), protein C inhibitor (2HI9), plasminogen activator inhibitor-1 (1B3K), C1-inhibitor (5DU3), antithrombin (1T1F), neuroserpin (3FGQ), serpins from insects and trematodes *Manduca sexta* (1K9O), *Anopheles gambiae* (3PZF), *Tenebrio molitor* (3OZQ), *Schistosoma mansoni* (3STO), the plant *Arabidopsis thaliana* (3LE2), and the bacteria *Thermobifida fusca* (1SNG) and *Thermoanaerobacter tengcongensis* (2PEE). An extract of the alignment adjacent to residues 56, 114, 116, 186 and 334 is shown, with colours denoting non-polar (yellow), polar (green), glycine/proline (orange), acidic (red) and basic (blue) amino acids. (**c**) Following minimization and equilibration, a 50 ns molecular dynamics simulation was conducted for the wild-type protein and mutant using NAMD. The root-mean-square distance between the α-carbon atoms of residues at positions 56, 186 and 334 at each frame in the resultant trajectories was calculated using VMD, showing a marginal compaction of this triad in the mutant (red) upon loss of interactions coordinated by the wild-type asparagine side-chain (blue), but no gross distortion due to incorporation of the bulky tyrosine side-chain.

In the context of the three-dimensional structure of AAT, the N186Y substitution of the Bologna variant occurs in the central region of β-sheet A, termed the ‘shutter’ (Figure 1a, top left panel). The shutter was first identified from the observation of a cluster of aggregation-prone mutations arising in several members of the serpin family including antithrombin [27]. Asparagine 186 is situated on β-strand 3A and plays a central role in a network of side-chain-mediated hydrogen bonds: this residue forms a bond with the adjacent H334 on β-strand 5A, as well as to S56 on helix B and N116 on β-strand 2A (Figure 1a, top right panel (i)). The presence of such a network of hydrogen bonds is a feature common to serpins, and both H334 and N186 are highly conserved throughout the superfamily (Irving et al., 2000) (Figure 1b). The importance of H334 to this network has been highlighted by the tendency of point mutations at this site to result in polymerogenic behaviour [38,40]. Most strikingly, H334 and N186 straddle the central region of β-sheet A that accommodates an inserting reactive centre loop during protease inhibition or polymerization. In order to undergo this canonical conformational change, β-strands 1A-3A translate laterally away from β-strands 5A-6A, requiring the H334-N186 hydrogen bond to be broken in the process (Figure 1a, lower left and lower right panel (iii)). Therefore, this bond provides some contribution to the energetic barrier that limits the opening of β-sheet A [40].

When the N186Y substitution is introduced into the native protein, it is evident that the bulky polar tyrosine side-chain should be readily accommodated by an adjacent cavity through the displacement of two ordered water molecules that are observed in the crystal structure (Figure 1a, black arrows in the middle right panel (ii)). Furthermore, while the new side-chain is unable to maintain the hydrogen bond between positions 186 and 116—representing a link between β-strands 2A and 3A—the hydroxyl group of the side-chain is predicted to substitute directly for one of these displaced water molecules, forming a hydrogen bond to the γ-oxygen atom of threonine 114 and thus maintaining a link between these strands. These factors are expected to ameliorate to some extent the molecular consequences of this non-conservative substitution (Figure 1c). However, the N186 bond with S56 and, critically, with the H334 side-chain are both lost in this scenario (Figure 1a, red arrows in the upper right panel (i)). As the latter interaction contributes to the stability of the association between β-strands 3A and 5A, this would decrease the energetic penalty to opening of β-sheet A in this variant, and thereby be expected to result in an increased tendency to polymerize.

Thus, the deficiency state of individuals with the Bologna variant is likely the result of a molecular defect, introduced into a conserved and functionally important region, that is expected to increase the ability of this protein to undergo pathological conformational change associated with polymerization. To test this hypothesis and characterize this variant in a physiologically relevant system, we next explored its behaviour in cellular models of disease.

### 2.3. Intracellular Polymerization and Secretion Deficiency of the Bologna Variant Expressed in a Hepatoma Cell Line

In order to investigate the effects of the new mutation on the intracellular handling and secretion of AAT, the Bologna AAT variant was expressed in the liver-derived Hepa 1.6 cell line, as previously used for other rare deficiency AAT variants [44,45,46,47]. The Bologna mutant was compared to wild-type M AAT, the common polymerogenic breach-region Z AAT variant (Glu342Lys) and the rare shutter region deficiency variants King’s (His334Asp) and Mmalton (Phe51del) AAT. Polymer formation was first assessed by the tendency of the variants to accumulate as intracellular aggregates not solubilized by NP-40 treatment. As recently described [20], the partitioning of intracellular AAT between soluble and insoluble fractions after lysis in the presence of non-ionic NP-40 detergent at 1% *v*/*v* concentration represents a simple and reliable method to assess the tendency of AAT variants to polymerize and accumulate within inclusion bodies.

**Figure 2:**
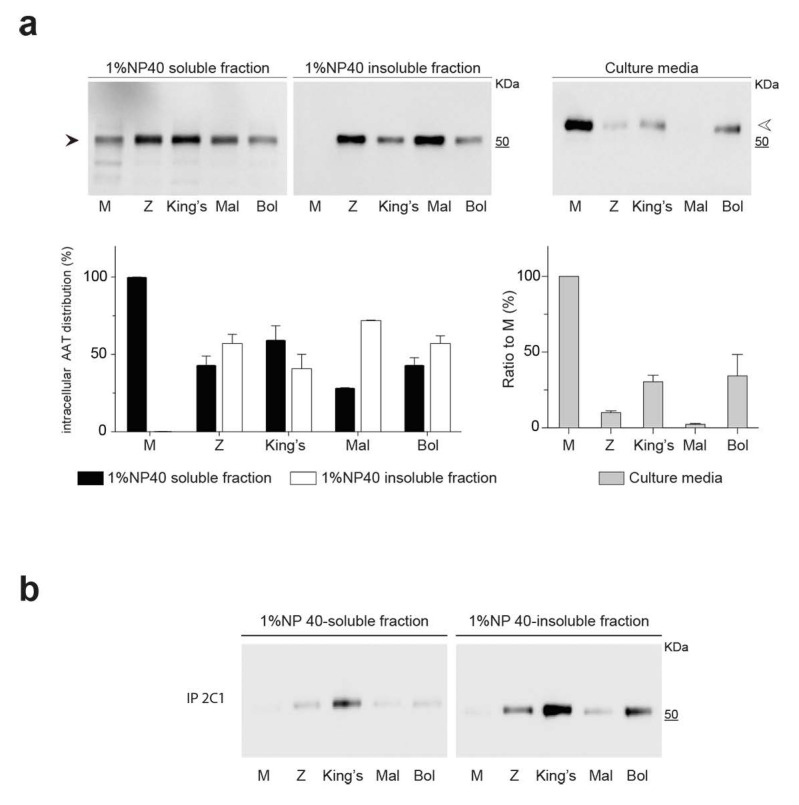
Cellular handling of Bologna AAT. (**a**) Hepa 1.6 cells were transfected to express wild-type M, Bologna (Bol) or the polymerogenic AAT variants Z, Mmalton (Mal) and King’s. Forty-eight hours after transfection, the culture media were collected, and the cells were lysed in 1% *v*/*v* NP-40 buffer. The NP-40-soluble and -insoluble cellular fractions and the cell media were separated by 4–12% *w*/*v* acrylamide SDS-PAGE and AAT was detected by immunoblotting with a polyclonal antibody (Dako). Black and white arrowheads indicate high-mannose and complex *N*-glycosylated forms of AAT, respectively. AAT levels in the two cellular fractions (expressed as percentage of total intracellular AAT) and in the cell media (expressed as percentage of secreted M AAT) were determined by densitometric quantification and reported in the graphs as mean ± SEM (*n* = 3). (**b**) Soluble and insoluble intracellular fractions prepared as in panel A were immunoprecipitated using the AAT polymer-specific 2C1 mAb and analyzed by 4–12% *w*/*v* acrylamide SDS-PAGE followed by immunoblotting for total AAT.

Hepa 1.6 cells, transiently transfected to express the AAT variants, were lysed in a buffer containing 1% *v*/*v* NP-40 and centrifugation at 16,000× *g* was used to separate the NP-40-soluble and -insoluble fractions. The latter was mechanically resuspended in the same volume of NP-40-buffer and sonicated to release the insoluble components. The distribution of AAT in the intracellular fractions was analyzed by SDS-PAGE and immunoblot, followed by densitometric quantification of the AAT bands (Figure 2a). As expected for a readily secreted protein, M AAT was exclusively present in the detergent-soluble fraction, while polymerogenic Z AAT was distributed approximately equally between the NP-40-soluble and -insoluble fractions. Likewise, the Bologna, King’s, and Mmalton mutants showed partial accumulation as detergent-insoluble intracellular aggregates. Immunoblot analysis of the AAT variants in cell culture media showed severe secretory deficiency for the mutant proteins, with extracellular levels of 10.0 ± 1.2, 30.4 ± 4.3, 2.3 ± 0.59 and 34.3 ± 14.1% (±SEM) of the M AAT level for Z, King’s, Mmalton, and Bologna AAT, respectively. All variants in the culture media showed a slower electrophoretic migration, in agreement with glycosylation changes after transiting the Golgi apparatus, indicating minimal release due to cell death.

The accumulation of polymers was further assessed by immunoprecipitation of the intracellular fractions with the 2C1 monoclonal antibody (mAb), a conformational antibody that specifically binds to AAT polymers [38] (Figure 2b). The 2C1 mAb did not precipitate material from any of the cellular fractions of M AAT, confirming the monomeric state of wild-type AAT and the specificity of the mAb under the expression conditions used in our assays. Conversely, Z, King’s, and Bologna AAT polymers were immunoprecipitated by mAb 2C1 from both the soluble and insoluble intracellular fractions. Notably, Mmalton AAT, which showed accumulation in the detergent-insoluble fraction (Figure 2a), was immunoprecipitated less efficiently by 2C1 mAb compared to the other mutated variants.

### 2.4. Detection of Bologna AAT Polymers in Cell Culture Media and Plasma

**Figure 3:**
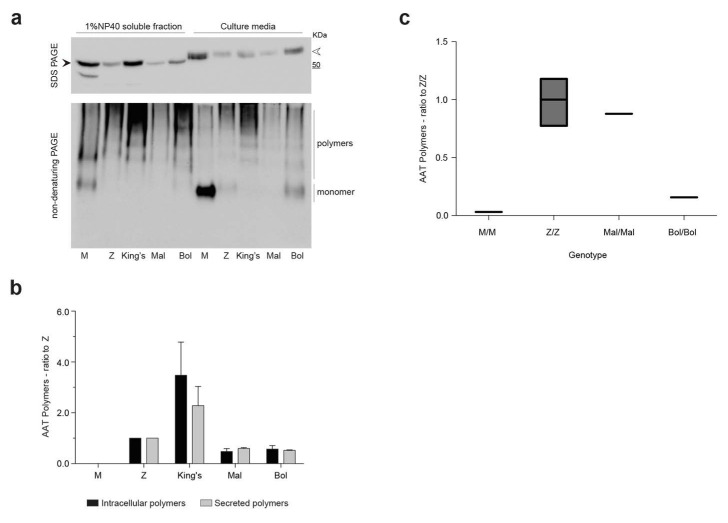
Intra- and extracellular polymers of the Bologna AAT variant. (**a**) HEK293T cells were transfected to express wild-type M AAT or the indicated AAT variants. Forty-eight hours after transfection, the cell culture media were collected, and the cells lysed in 1% *v*/*v* NP-40 buffer. All samples were separated by 10% *w*/*v* SDS-PAGE (upper panel) or 8% *w*/*v* non-denaturing PAGE (lower panel), and AAT was detected by immunoblotting with a polyclonal antibody (Dako). (**b**) The same samples analyzed in panel A were tested by sandwich ELISA using the 2C1 mAb to capture AAT polymers only. The graph shows mean ± SEM (*n* = 2) of the concentration of intracellular (black bars) and secreted (grey bars) polymers, calculated by using a standard curve and normalized to polymer levels in the Z AAT samples. (**c**) Quantification of AAT polymers in the plasma of a PI*Bologna/Bologna AAT homozygous patient. Polymers were quantified by ELISA using the 2C1 mAb for capture, and including plasma samples from one PI*Mmalton/Mmalton, three PI*Z/Z and two PI*MM subjects as references.

To further characterize polymer formation and secretion, the Bologna variant was expressed in the HEK293T cell line, which allows higher levels of protein expression than Hepa 1.6 cells. Following transfection, M AAT and the mutated variants Z, King’s, Mmalton and Bologna in the NP-40-soluble cellular lysates were analyzed by immunoblot after separation by either SDS-PAGE (Figure 3a, upper panel) or non-denaturing PAGE (Figure 3a, lower panel). The behaviour of all variants in SDS-PAGE was consistent with that observed in Hepa 1.6 cells (Figure 2a). Under non-denaturing conditions, M AAT migrated mainly as monomer (Figure 3a, bottom panel), with the intracellular high-mannose *N*-glycosylated form migrating marginally more slowly than the extracellular form, which would have undergone glycan maturation in the Golgi (white arrowhead). High molecular weight complexes, with the typical ladder profile of polymeric AAT, were detected intracellularly for the control polymerogenic variants and for Bologna AAT. Extracellularly, the Z and Bologna AAT variants showed both monomeric and polymeric forms, while the King’s variant was exclusively present as polymers, in agreement with previous reports showing a high polymerogenic tendency for this mutant [38]. Mmalton AAT was weakly detected as polymeric ladders both in the soluble intracellular fraction and in the culture medium, in agreement with a high proportion of this mutant protein accumulating in the insoluble fraction, as seen in Hepa 1.6 cells.

The presence of polymers for each AAT variant in the soluble intracellular and secreted fractions was evaluated by a sandwich ELISA using mAb 2C1 as the capture antibody (Figure 3b). This approach allowed us to quantify the polymers as observed by non-denaturing PAGE in Figure 3a and confirmed the high tendency of King’s AAT to form soluble polymers, while lower levels were observed for Z AAT and the other shutter domain variants.

We then investigated whether polymers could also be identified in the plasma, again using an ELISA with capture by mAb 2C1. The plasma polymer content was determined for patient 2.1 (PI*Bologna/Bologna, Table 1) in comparison with a PI*Mmalton/Mmalton patient as well as with PI*MM and PI*ZZ control samples. All the subjects were non-smokers and none was receiving augmentation therapy. As shown in Figure 3c, only background signal was seen in the MM control samples, and the assay was therefore unaffected by the presence of AAT monomers. Compared to the polymer content observed in the ZZ plasma samples, the Mmalton/Mmalton sample showed a slightly decreased level, while that detected in the Bologna/Bologna sample was lower. The secretion of Bologna polymers in our cellular models of AATD is therefore consistent with the behaviour of this variant in vivo.

### 2.5. Assessment of Intracellular Polymers of Bologna AAT by Immunofluorescence and Confocal Microscopy

**Figure 4:**
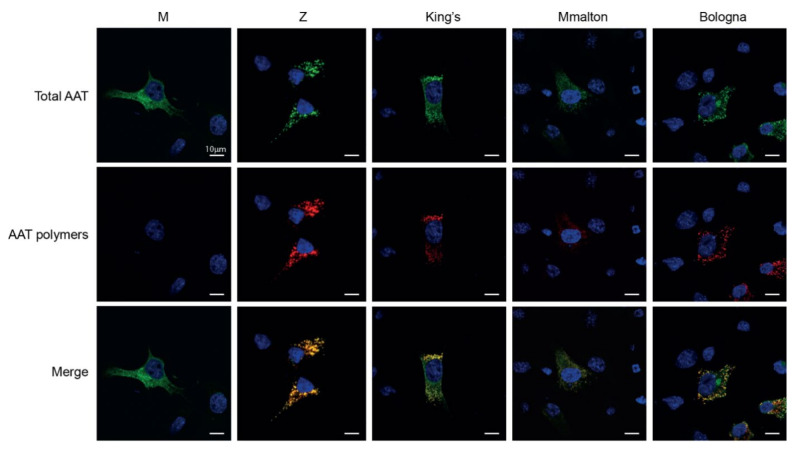
Mutant variants of AAT accumulate intracellularly as 2C1-positive polymers. Hepa 1.6 cells seeded on glass coverslips were fixed 48 h after transfection with the indicated AAT variants. After permeabilization, cells were immunostained with an anti-human AAT polyclonal Ab (Dako) (green) or with the anti-AAT polymers 2C1 mAb (red). Merged panels are shown with overlapping signals in yellow. Nuclei were stained blue by the Hoechst dye. Cells expressing Z, King’s, Mmalton and Bologna AAT variants showed a punctate pattern of 2C1-positive polymers. Scale bar in all panels is 10 μm.

Expression of Z AAT in cells results in the formation of punctate structures with their origin in the ER, consistent with the accumulation of inclusion bodies in the liver [48]. To determine whether this is also observed with the new shutter region mutant, Hepa 1.6 cells expressing the variants were characterized by immunofluorescence and confocal microscopy. These cells were transfected with constructs encoding the different variants, fixed after 48 h and co-stained with a non-conformation-selective polyclonal antibody and the polymer-specific 2C1 mAb (Figure 4). As expected, wild-type M AAT was only recognised by the polyclonal antibody, with a reticular staining pattern typical of a secretory protein contained within the ER, as seen before in this [45,49] and other cellular models of AAT deficiency [38]. In contrast, all AAT mutant variants produced a pattern of intense, punctate inclusions with colocalization of the signals of the anti-total and anti-polymer antibodies, presumably associated with the ER, as shown before for Z and King’s AAT [19,38,48].

### 2.6. Kinetics of Accumulation and Secretion of the Bologna AAT Variant

**Figure 5:**
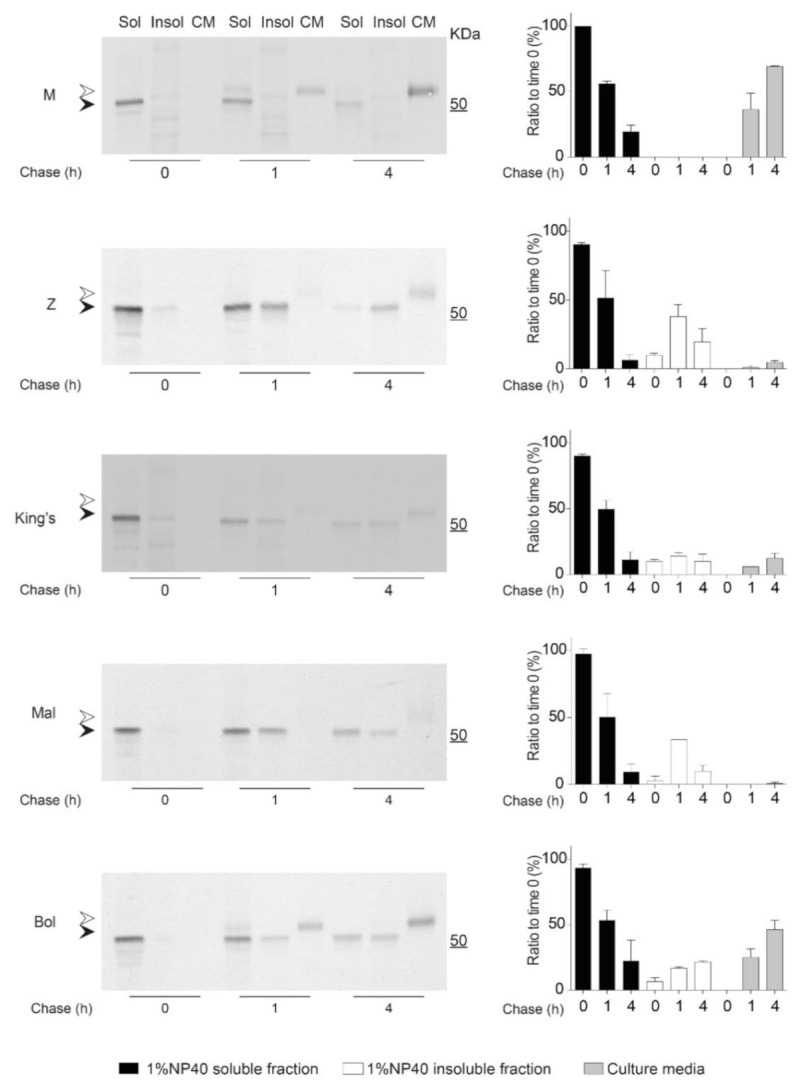
Kinetics of intracellular accumulation and secretion of AAT variants. Hepa 1.6 cells were transfected with the AAT variants as indicated. After 48 h of expression, the cells were labelled for 10 min with ^35^S-Met/Cys and chased for the indicated times. Culture media were collected, and cells were lysed in 1% *v*/*v* NP-40 buffer for preparation of the soluble and insoluble intracellular fractions. All samples were immunoprecipitated with an anti-AAT polyclonal Ab (Dako). Samples were resolved in a 4–12% *w*/*v* acrylamide SDS-PAGE and detected by autoradiography. White and black arrowheads indicate the complex and high-mannose glycosylated forms of AAT, respectively. Autoradiograms from two independent experiments were quantified by densitometry to determine AAT levels, using the Image Studio Lite software (LI-COR Biosciences, Cambridge, UK). Graphs show mean ± standard error of the mean of radioactive AAT normalized to the t = 0 sample for each variant (*n* = 2).

Protein folding and aggregation are dynamic, kinetic processes, and as such relative differences in misfolding, deposition, degradation and secretion can have pronounced effects on molecular fate [20,50]. To investigate the kinetics of intracellular polymer accumulation and their consequences for secretion, we performed pulse-chase experiments on Hepa 1.6 cells transfected with the panel of AAT variants. Cells were pulse-labelled for 10 min with ^35^S-methionine and cysteine, and chased for 0, 1 and 4 h. Culture media and NP-40-soluble and insoluble fractions, obtained as described above, were immunoprecipitated with an anti-human AAT polyclonal antibody and resolved by SDS-PAGE. M AAT (Figure 5, M panel and graph) was readily detected in the soluble intracellular fraction at the 0 h time point as an immature form close to the 50 kDa marker (Figure 5, M panel, black arrowhead), was not present in the insoluble fraction, and after 1 h of chase it was detected in the culture medium as a fully glycosylated slower-migrating form (Figure 5, upper panel, white arrowhead). In contrast, Z AAT (Figure 5, Z panel and graph) was poorly secreted and detected at every time point in both intracellular fractions, with a significant amount contained within the insoluble aggregates.

The analysis of the other polymerogenic variants (Figure 5, corresponding panels and graphs) demonstrated different extents of intracellular accumulation and secretion. King’s AAT was characterized by a decreased deposition into the insoluble intracellular fraction and increased secretion levels compared to Z AAT, in agreement with our steady-state analysis by immunoblot (Figure 2). Mmalton AAT showed a pattern of intracellular accumulation similar to Z AAT, but with the lowest amount of AAT secretion. Bologna AAT, instead, showed a milder tendency to accumulate and a moderate secretion defect. Our pulse-chase analysis also showed, for each AAT variant, that not all of the radiolabelled AAT protein present at the initial time was recovered after the 4 h chase, suggesting a degree of intracellular protein degradation.

## 3. Discussion

Rare variants have collectively provided an invaluable source of information on the molecular mechanisms underpinning physiological and pathological conformational change among serpins. The identification of a cluster of deficiency mutations of antitrypsin, antithrombin, anti-chymotrypsin and C1-inhibitor revealed the crucial role of the serpin shutter region of β-sheet A in regulating these conformational changes [27] and this has since been noted among other members of the family including neuroserpin [51,52].

In this work, we report a novel shutter region mutant of AAT associated with severe plasma deficiency and evidence of pulmonary disease in patients. In the first proband, which showed very low AAT plasma levels and severe pulmonary manifestations, an allele encoding a N186Y mutation was present in compound heterozygosity with Z AAT. Dubbed AAT Bologna, subsequent detection of this allele was made in four further unrelated probands. The clinical data of the adult patients corroborated the protein deficiency of the N186Y variant and its pathological consequences, as these individuals exhibited lung disease apparently exacerbated by smoking and environmental exposure. The different geographical origin of the probands, namely Italy, Egypt, and Ukraine, did not support a founder effect of the N186Y allele, and tentatively suggested distribution of this variation in the vicinity of the Mediterranean and the Black Sea. Although evidence of liver manifestations in the patients was limited, localization of the mutation in the shutter region, given its association with a number of polymerization-prone AAT variants, including Mmalton/Mpalermo, Siiyama, King’s, Baghdad [32,35,38,53], and neuroserpin variants S52R and H338R [51,52], prompted us to further investigate the molecular mechanism of the Bologna variant in cellular models, with reference to other known AAT mutants.

Consistent with other pathogenic variants involving perturbation of the shutter region, our results showed that the Bologna mutation causes intracellular polymer formation of the 2C1 mAb-positive type, as seen before for Z, King’s, and Siiyama variants [38]. This suggests, in turn, that Bologna forms polymers through a C-terminal domain-swap [14,15] and distinguishes it from an alternative polymerization mechanism believed to be exhibited by the rare Trento variant [46]. As expected for mutant forms of AAT that undergo polymerization, Bologna AAT partially partitioned into the insoluble intracellular fraction, in conjunction with a moderate degree of secretion deficiency from cultured cells compared to Z and Mmalton AAT. Secretion levels of AAT Bologna were instead similar to those of the King’s variant, with the difference that extracellular King’s AAT was entirely polymeric, while Bologna AAT also yielded a monomeric component.

Secretion deficiency for Z AAT arises from accumulation of polymeric material and from intracellular degradation by ERAD of a substantial fraction of the synthesized protein [12,13]. The incomplete recovery of radioactivity during the pulse-chase experiments performed here indicates that a proportion of AAT Bologna was also likely impacted by this process. The presence of polymeric Bologna AAT in the culture medium is likely the result of active secretion, as seen previously with Z AAT [19].

The Bologna mutation alters a highly conserved position that, in the native state of AAT, coordinates a network of hydrogen bonds to H334, S56 and N116. During reactive centre loop insertion into β-sheet A, there is a concerted movement of the two halves of the sheet with respect to one another; in the process, interactions between strands 3A and 5A must be disrupted, and new ones formed upon incorporation of the loop as strand 4. While the residues associated with the shutter are buried into the hydrophobic core of the molecule, a location that would generally favor non-polar side-chains, this inward-facing histidine–asparagine–serine network makes an important contribution to stability in this region that is broken when the reactive centre loop is cleaved by a protease or during polymerization (Figure 1a, panels (i) and (iii)). The H334 residue has been recognized in several studies as contributing to the stability of the native (or a native-like) structure against inappropriate conformational change [38,40,52,54], and selection at S56 is substantial enough that an interchange between the two clusters of serine codons, TCN and AGY, can be traced back to an evolutionary event that occurred between divergence of protostomes from deuterostomes [55]. While the bulky hydrophobic ring of the tyrosine in the Bologna mutant appears to be sufficiently accommodated by its proximity to a water-filled pocket, the true defect the mutant introduces is the loss of these coordinating bonds (Figure 1a, panel (ii)). The characterization of the variant performed here extends our understanding of the importance of this network, with evident evolutionary constraints reflected by a very high degree of conservation on N186 throughout the serpin superfamily [56], and the striking dichotomy between two polar amino acids, histidine and glutamine, at position 334, reflecting a marked negative selective pressure on both of these sites (Figure 1b).

Taken together, our observations of the behaviour of the Bologna variant in cells are consistent with its low levels in plasma coupled with the presence of circulating polymers. The Bologna mutation is therefore a key tool for interrogating the interactions within the shutter domain, alongside established variants Mmalton, Siiyama, and King’s.

## 4. Materials and Methods

### 4.1. Genetic Analyses and Clinical Data

Biochemical and genetic tests to diagnose AATD were performed at the Centre for Diagnosis of Inherited Alpha1-Antitrypsin Deficiency in Pavia (Italy) with the understanding and written consent of each subject. All methodologies were in accordance with the Declaration of Helsinki and were approved by the local ethics committee. The plasma levels of AAT and C-reactive protein were determined by a rate immune nephelometric method assay (Immage Immunochemistry System; Beckman-Coulter, Milan, Italy). DNA was isolated from whole peripheral blood using a commercial extraction kit (QIAmp DNA Blood Minikit on QiaCube, Qiagen, Milan, Italy). Genotyping for detection of the S and Z allelic variants was performed by PCR with fluorescently labelled Taq-Man probes (Vic or Fam labels) on a LigthCycler 480 (Roche Diagnostics, Monza, Italy) [57]. The new mutation was identified by sequencing all coding exons (II-V) of the *SERPINA1* gene (RefSeq: NG_008290) as reported previously [58], using the CEQ 8800 genetic analysis System (Beckman Coulter, Milan, Italy). The clinical data were obtained from direct observation of clinical charts and they are reported in an anonymized form. Clinical data presented here are part of Italian Registry of Alpha 1-antitrypsin Deficiency (RIDA1), that received ethical approval by IRCCS Policlinico S. Matteo, Pavia (Italy) on 14 January 2019 (n°0385).

### 4.2. Expression Vectors

Expression vectors encoding M1(Val213), Z, King’s and Mmalton AAT are based on plasmid pcDNA3.1 and have been described previously [23,38,45]. The vector encoding the Bologna variant was obtained by site-directed mutagenesis of the M-encoding plasmid using the QuikChange II Site-directed mutagenesis kit (Agilent, Milan, Italy), according to the manufacturer’s protocol, using primer 5′gacacagtttttgctctggtgtattacatcttctttaaaggca and its complement.

### 4.3. Cell Culture and Transfection

Hepa 1.6 is a mouse hepatoma cell line (ATCC CRL-1830) and HEK293T is a human embryonic kidney cell line (ATCC CRL-16268). Both cell types were cultured in Dulbecco’s modified Eagle medium (DMEM) supplemented with 10% *v*/*v* fetal bovine serum. Transient transfections of Hepa 1.6 or HEK 293T cells were performed with polyethyleneimine ‘MAX’ (PEI) (Polysciences, Hirschberg, Germany) and plasmids encoding the AAT variants. In brief, for a 9 cm^2^ well, 20 µg PEI and 3 µg plasmid were incubated for 20 min in 400 µL serum-free DMEM, diluted to 1.1 mL with complete culture medium, added to the cell layer and incubated for 5 h. Transfected cells were then washed twice with pre-warmed PBS and further incubated at 37 °C with 1 mL of Opti-MEM (Gibco, Thermo Fisher Scientific Ltd., Loughborough, UK). After 20 h incubation, cell culture media were collected, centrifuged at 800× *g* for 5 min at 4 °C, transferred into clean tubes and stored at −20 °C, while the cells were lysed as described below.

### 4.4. Cell Lysis and Fractionation

Both Hepa 1.6 and HEK293T transfected cells were lysed at a concentration of 2.5 × 10^6^ cells·mL^−1^ in 1% *v*/*v* NP-40 buffer (10 mM Tris, pH 7.4, 300 mM NaCl, 1% *v*/*v* NP-40) supplemented with protease inhibitors (Roche Ltd., Hertfordshire, UK). Cell lysates were collected and mixed for 30 min at 4 °C on a rotator mixer. 1% *v*/*v* NP-40-insoluble and NP-40-soluble fractions were separated by centrifugation at 16,000× *g* for 15 min at 4 °C. The supernatant was collected (1% *v*/*v* NP-40-soluble fraction), while the pellet (1% *v*/*v* NP-40-insoluble fraction) was washed twice in 1% *v*/*v* NP-40 buffer and mechanically resuspended in an equal volume of 1% *v*/*v* NP-40 buffer supplemented with protease inhibitors. The 1% *v*/*v* NP-40-insoluble fraction was finally solubilized by sonication at 1.15 KHz (5 µm amplitude) for 15 s at RT (Soniprep 150; MSE Ltd., London, UK).

### 4.5. SDS-PAGE, Non-Denaturing PAGE and Immunoblots

Samples analyzed by SDS-PAGE were resolved on pre-cast NuPAGE™ 4–12% *w*/*v* acrylamide Bis/Tris Protein Gels (Invitrogen, Thermo Fisher Scientific Ltd., Loughborough, UK) and transferred to LF-PVDF membranes (Millipore Ltd., Hertfordshire, UK). Membranes were saturated in 5% *w*/*v* low-fat milk (Cell Signaling Technology, Danvers, MA, USA) in PBS-0.1% *v*/*v* Tween, probed with anti-human AAT polyclonal antibody (Dako, Agilent, Stockport, UK) followed by horseradish peroxidase (HRP)-conjugated secondary antibodies (Santa Cruz Biotechnology, Dallas, TX, USA), and finally revealed by ECL (Clarity; Bio-Rad Laboratories, Watford, UK). Western blot images were acquired with an Image Quant Las400 (GE Healthcare Life Sciences, Amersham, UK) and analyzed using Image Studio Lite software (LI-COR Biosciences, Cambridge, UK). For non-denaturing PAGE, we followed a procedure previously reported for serpin polymers [51,59]. Briefly, the samples were mixed with non-denaturing loading buffer (without β-mercaptoethanol and SDS), analyzed with 7.5% *w*/*v* acrylamide gels made in-house, and transferred to PVDF membranes in the absence of methanol, followed by immunoblot as described above.

### 4.6. Sandwich ELISA

Polymeric AAT was quantified by sandwich ELISA following the basic protocol previously described [38], with the following modifications: the capture antibody was purified 2C1 mAb at 2 µg/mL. An AAT polymer standard was prepared by heating purified M AAT 0.5 mg/mL at 55 °C for 16 h; bound polymers were detected with mAb 3C11 [16] conjugated to HRP following the manufacturer’s protocol (Lightning-Link^®^ HRP Conjugation Kit, Expedeon|Abcam, Cambridge, UK) or, for plasma polymer detection, by a commercial HRP-conjugated sheep anti-AAT polyclonal antibody (Abcam, Cambridge, UK).

### 4.7. Pulse-Chase Experiments

Hepa 1.6 cells transfected with plasmids encoding the AAT variants were pulse-labelled 48 h after transfection with ^35^S-Cys/Met (EasyTag™ Express Protein Labelling; Perkin Elmer, Beaconsfield, UK), 0.45 MBq/10^6^ cells for 10 min in DMEM without Cys/Met, and then chased in complete culture medium for 0, 1 and 4 h. For every chase time point, 1% *v*/*v* NP-40-soluble and -insoluble intracellular fractions and cell media were collected as previously described. Radiolabelled AAT was isolated by immunoprecipitation with a polyclonal anti-human AAT antibody (Dako, Agilent, Stockport, UK) and resolved by SDS-PAGE as described above, followed by autoradiography. Densitometric analysis of AAT bands was performed with Image Studio Lite (LI-COR Biosciences, Lincoln, NE, USA).

### 4.8. Immunofluorescence and Confocal Microscopy

Hepa 1.6 cell were seeded onto 2 cm^2^ coverslips (Millipore Sigma) and transfected as described above. After 48 h, cells were fixed with 4% *v*/*v* paraformaldehyde, permeabilized with 0.1% *v*/*v* Triton X-100, and immunostained with anti-human AAT (Dako, Agilent, Stockport, UK) (2.2 μg/mL) or the anti-AAT polymer 2C1 mAb (0.8 μg/mL) overnight at 4 °C. The primary antibodies were respectively detected with goat anti-mouse antibody conjugated to Alexa Fluor 488 and goat anti-rabbit antibody conjugated to Alexa Fluor 555 (Thermo Fisher Scientific, Loughborough, UK), respectively. Cells were counterstained with Hoechst (Thermo Fisher Scientific, Loughborough, UK) to visualize the nuclei. Coverslips were mounted on slides with Immuno-Mount (Thermo Fisher Scientific, Loughborough, UK) and analyzed on a Zeiss LSM700 confocal microscope with a 63× objective (1.4 oil).

### 4.9. Molecular Modelling, Dynamics and Sequence Analysis

The predicted structural effect of the N186Y substitution was evaluated by in silico mutagenesis of the native structure obtained as PDBe database (https://www.ebi.ac.uk/pdbe; acessed on 1 February 2021) entry 3NE4 [41]. Examination of the associated electron density map provided by the PDBe server showed the clear presence of additional unbuilt ordered water molecules including one in the vicinity of N186. Therefore, using the deposited structure factors, a single round of PHENIX refinement with automatic placement of water molecules [42] was performed on the coordinates. The tyrosine substitution was then made using Coot [60], requiring the removal of two water molecules and the replacement of one by the hydroxyl group of the introduced side-chain. These modified coordinates were then also refined in PHENIX to permit any adjustment of neighboring residues required to minimize steric clashes, with restraints placed on the backbone atoms at position 186 and occupancy of its side-chain decreased to 0.01 to minimize the weighting against the asparagine electron density. The resulting models were visualized using ChimeraX [43].

To evaluate the stability of the geometry of the H334-Y186-S56 triplet with respect to the wild-type H334-N186-S56 residues, molecular dynamics simulations were performed using NAMD3 [61] and analyzed using VMD [62]. Missing residues at the *N*-terminus of the two models described above were added using Coot as a region extended away from the experimentally defined molecule. The models were placed in solvent (TIP3P model) using a box margin of 14 Å in all directions with the overall protein charge neutralized by sodium ions and with parameterization by the CHARMM36 forcefield [63]. Energy minimization (1 × 10^5^ steps; periodic boundary conditions; particle mesh Ewald electrostatics; short-range non-bonded interaction cut-off smoothly between 10 Å and 12 Å; pair-list cut-off 14 Å) was confined first to the solvent, then extended to all atoms. Following this, the system was equilibrated using these parameters for 10 ns to 310 K and simulated for 50 ns with a Langevin thermostat (damping coefficient of 5/ps) and Nosé-Hoover Langevin barostat using an NPT ensemble at 1 atm, rigid covalent hydrogen bonds maintained using the SHAKE algorithm for protein and the SETTLE algorithm for solvent, and a 1 fs timestep.

For the comparative sequence analysis, the crystal structures of several serpins in the native conformation were obtained from the PDB (http://www.rcsb.org; accessed on 1 February 2021), aligned using MUSTANG [64], and the structure-based sequence alignment viewed using MEGA X [65].

## 5. Conclusions

The serpin shutter region is central to the β-sheet-opening mechanism of the serpin fold. Within this region, three highly conserved positions—56, 186, and 334 in alpha-1-antitrypsin numbering—form polar contacts that straddle the site of reactive centre loop insertion during protease inhibition and polymerization. The identification of a naturally arising pathogenic mutation in alpha-1-antitrypsin, N186Y, that we have named Bologna, reveals the importance of this residue to the proper formation and release of a monomeric molecule. Cellular models show that, in the absence of the interactions mediated by the asparagine side-chain, alpha-1-antitrypsin has a tendency to form polymers that contribute to a severely deficient state. In combination with other shutter domain variants, including Mmalton, Siiyama, and King’s, these results extend our understanding of the importance of this buried network of polar residues.

## Figures and Tables

**Table 1:** Proband carriers of the Bologna variant and their families.

Code	Genotype	AAT ^1^	CRP ^2^	Age ^3^	Clinical Presentation
1.1. Proband	Z/Bologna	0.23	0.001	50	Emphysema, bronchiectasis, diabetes, liver steatosis
1.2. Son	M1/Z	0.67	0.007	16	Healthy
1.3. Daughter	M1/Bologna	1.49	0.009	14	Healthy
2.1. Proband	Bologna/Bologna	0.34	0.004	44	Emphysema
2.2. Son	M1/Bologna	0.60	0.001	14	Healthy
2.3. Daughter	M1/Bologna	0.72	0.001	8	Healthy
3.1. Proband	Bologna/Bologna	0.28	0.003	39	Emphysema
4.1. Proband	M1/Bologna	0.95	0.006	74	Emphysema, chronic bronchitis
5.1. Proband	M1/Bologna	0.86	0.001	58	Hepatitis

^1^ Concentration of plasma AAT (normal values are 2.00–0.9 g/L).^2^ Concentration of plasma CRP (normal values are <0.008 g/L). ^3^ Age at diagnosis.

## Data Availability

All the data required to evaluate the findings of the authors are found within the manuscript or, in the case of the third-party crystal structures used, in the PDBe database (https://www.ebi.ac.uk/pdbe/; acessed on 1 February 2021).
